# Supplementary figures and images for: Effects of Trehalose Preconditioning on H9C2 Cell Viability and Autophagy Activation in a Model of Donation after Circulatory Death for Heart Transplantation
Source: Curr Issues Mol Biol. 2024 Apr 12;46(4):3353–63. doi: 10.3390/cimb46040210 (PMC11049330; doi:10.3390/cimb46040210)

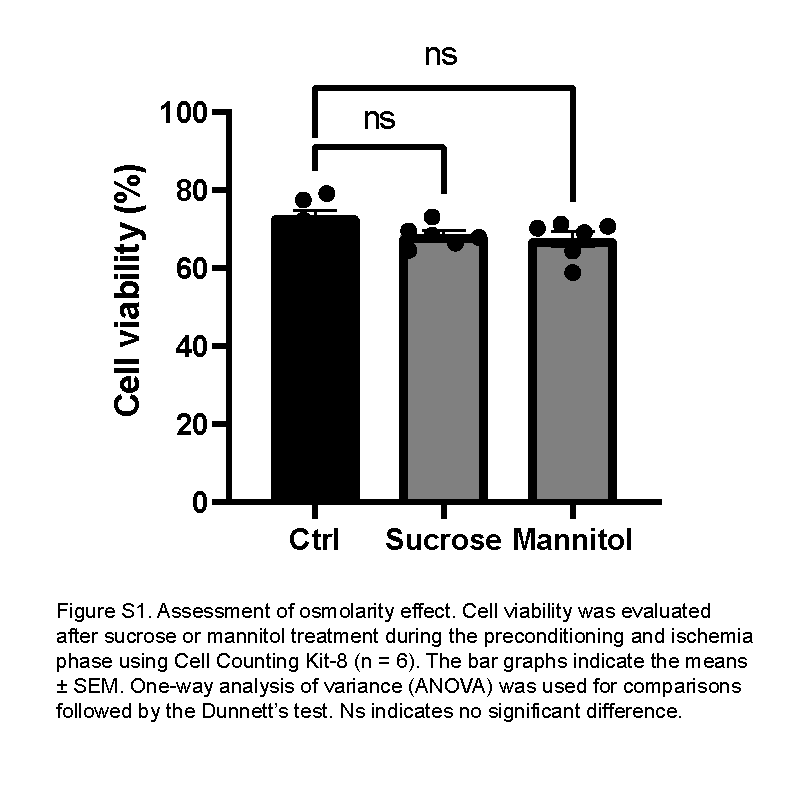

Supplement: Supplementary file 1 [file cimb-46-00210-s001.zip › cimb-2927154-supplementary.tif]
